# Age-Specific Seroprevalence of Anti-Hepatitis A Antibody Among 1-30 Years Old Population of Savadkuh, Mazandaran, Iran With Literature Review

**DOI:** 10.5812/hepatmon.6035

**Published:** 2012-05-30

**Authors:** Mohammed Jafar Saffar, Omid Abedian, Abolghasem Ajami, Farshideh Abedian, Araz Mohammad Mirabi, Ali-Reza Khalilian, Hana Saffar

**Affiliations:** 1 Pediatric Infectious Diseases Ward and Thalassemia Research Center, Bouali-Cina Hospital, University of Medical Sciences, Sari, IR Iran; 2 Molecular and Cellular Biology Research Center, Department of Microbiology and Immunology, Mazandaran University of Medical Sciences, Sari, IR Iran; 3 Department of Statistics, Mazandaran University of Medical Sciences, Mazandaran, Sari, IR Iran; 4 Department of Pathology, Shariati Hospital, Tehran University of Medical Sciences, Tehran, IR Iran

**Keywords:** Hepatitis A Virus, Antibodies, Seroprevalence, Iran

## Abstract

**Background:**

To determine age-speciﬁc seroprevalence rates of hepatitis A virus (HAV) immunoglobulin G (IgG) antibody in Savadkuh district, Mazandaran province, north of Iran, as well as to compare the collected data with earlier seroprevalence studies in the region and Iran in order to draw a proper epidemiological pattern for HAV infection in the country.

**Objectives:**

This study aimed to assess an age-speciﬁc HAV seroprevalence among 1- to 30-yearold people in Savadkuh, a less developed district of Mazandaran province, north of Iran.

**Patients and Methods:**

The study participants were 984 subjects who aged from one to 30 years and were residents of rural and urban areas of Savadkuh. They were selected using cluster sampling method and divided into ﬁve age groups: 1-2.9 (316 cases), 3-6.9 (254 cases), 7-10.9 (201 cases), 11-17.9 (115 cases), and 18-30 (98 cases). Anti-HAV antibody was measured by ELISA method. Seroprevalence rates among different age groups and their relationship to residency, educational levels of parents, water supply, and waste water disposal system was analyzed using chi-squared test.

**Results:**

Overall seroprevalence rate was 19.20 % with no signiﬁcant difference between rural and urban residents. The seroprevalence rates increased signiﬁcantly with age: from 5.7 % in age group 1-2.9 year to 34.8 % in adolescents, and to 68.4 % among young adults (P < 0.0001); regardless of signiﬁcant differences in educational levels among parents of residents in two areas it did not affect seroprevalence rates. Findings of this study and reviewing other reports from the region and the country suggest an epidemiological shift towards lower rates of anti-HAV antibody seroprevalence.

**Conclusions:**

It appears that anti-HAV antibody seroprevalence rate has been declining among Iranians and thereby more children would be susceptible to this infection. This would necessitate revising current strategies of preventative measures in Mazandaran and Iran.

## 1. Background

Hepatitis A virus (HAV) is an enterically- transmitted infection and leading cause of acute viral hepatitis throughout the world [1][2]. Epidemiologically, various geographical distribution of HAV infection exists that correlates closely with hygienic and sanitary conditions and other development indicators [3][4]. Clinical expression of HAV infection is highly age dependent and is minimal in children [1]. Children play an important role in HAV transmission. Distribution of anti-HAV antibody seroprevalence over age groups can be used as a marker for HAV epidemiologic pattern and viral transmission through a community [4][5]. Declining seroprevalence rate in a population, particularly in children, is an indicator to reduced incidence of HAV [1][4]. Many seroepidemiological findings revealed that a transition to lower rates of infection occurred in many hyperendemic countries within past two decades where their economic status improved [1][6]. In this situation, clinical manifestation of hepatitis A is likely to become a more serious problem in these countries. HAV infection could be prevented by immunization, and HAV vaccine may be used for pre-and post-exposure prophylaxis. Cost and feasibility are two major barriers to implementing HAV vaccination programs in these countries. Types of preventative strategies depend on epidemiologic characteristics of HAV infection and other vaccine-preventable diseases in any given country [7][12].

In Iran, no proper periodic and age-specific seroprevalence data are available nationwide and exact epidemiological characteristics of HAV infection are unknown. Results of earlier seroprevalence studies on different populations in the country suggested a hyperendemic pattern [13][14][15][16]. However, data collected in some recent studies demonstrated lower rates of infection especially among children [17][21].

## 2. Objectives

This study aimed to assess an age-specific HAV seroprevalence among 1- to 30-year-old population in Savadkuh, a less developed district of Mazandaran province, north of Iran. Also, in order to determine epidemiological characteristics of HAV infection in Mazandaran and over the country and to plan the most appropriate preventative strategies, relevant articles previously issued in Iran were reviewed.

## 3. Patients and Methods

This cross-sectional seroepidemiological study was conducted in Savadkuh district, Mazandaran province, north of Iran. Savadkuh is a less developed mountainous area of Mazandaran with 70,000 inhabitants, equally distributed between rural and urban areas. Its population density is 33/km(2) compared to 140/km(2) in Sari district, the capital of province. A river passes through almost all parts of the district. Act of agriculture is the main occupation of residents. In four regions, urbanization infrastructures were encouraged and more people moved to these regions known as city of Savadkuh.

Target population consisted of healthy 1- to 30-year-old residents of Savadkuh. Random cluster sampling was used to enroll required samples, which was proportional to the number of inhabitants in each area. Sample size calculation was performed based on a prevalence of 0.20 % with 0.025 precision and 95 % confidence interval which yielded a total 983 individuals. Epidemiologic data were collected from a questionnaire including age, sex, place of residence, educational levels of parents and adults, source of water supply, and method of waste water and sewage disposal. Based on education levels of adults and parents, the subjects were allocated into either of the following groups: I: illiterate-primary school, II: Junior secondary school, III: Senior secondary school and diploma, and IV: University educated levels. Also the participants were divided into five age groups: 1-2.9, 3-6.9, 7-10.9, 11-17.9, and 18-30 years. Study protocol was approved by the Ethics Committee of Mazandaran University of Medical Sciences. After written consent was obtained from parents and adults, blood samples were drawn. Sera were tested for anti-HAV antibodies using a qualitative ELISA method [HAV-Ab, DIA, Pro Diagnostic, Milano, Italy] according to the manufacturer’s instructions. The results were reported as positive or negative.

A descriptive analysis was followed by univariate analysis Carrying out qui-squared test to compare various subgroups with a 5% statistical significance levelOdds ratio and 95% confidence interval (CI) were calculated and presented for variables associated with risk of infections.

To determine epidemiological trends of HAV infection in Mazandaran and Iran, almost all relevant studies in Persian and English published in SID, Iran Medex, MEDLINE, Google, and Yahoo websites and containedkeywords HAV, Hepatitis A, HAV seroprevalence, HAV seroepidemiology, Mazandaran, and Iran were reviewed.

## 4. Results

All 984 individuals were enrolled in the study. The age groups and sex distribution, sites of residence, access to piped-water, and hygienic facility systems are shown in Table 1. As shown, there were no significant differences between rural and urban dwellers in regards to age, gender, and access to piped drinking water. In both areas (rural and urban), almost all households were employing enclosed defecation system but without any centralized waste water and sewage disposal systems. Table 1presents educational levels of the sample. The educational levels of parents and adults among urban dwellers were significantly higher than those of rural counterparts. The overall anti-HAV seroprevalence rate was 19.20 % with no statistically significant difference between urban and rural residents (18.4 % vs. 20.1 %, P = 0.4, OR = 0.89, CI: 0.65 - 1.23); Table 2. The seroprevalence rates increased significantly with age: from 5.7% in age group 1-2.9 year to 34.8% in adolescents, and to 68.4 % in young adults (P < 0.0001); Table 2. However, other variables including place of residence, educational levels, water supply, and waste water and sewage disposal systems did not influence anti-HAV antibody seroprevalence rates among rural and urban residents.

**Figure 1:**
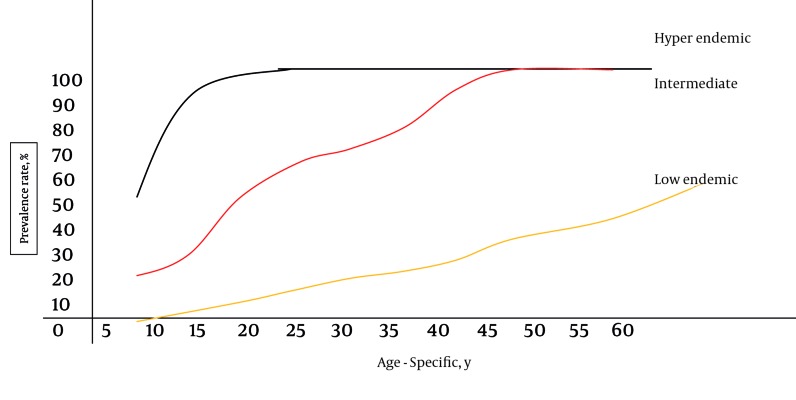
Different HAV Infection Patterns by Age-Specific Seroprevalence Rates (More than 90 %, 50 % and 5-10 % infection rates among > 15 years old population indicate high, intermediate, and low endemicity, respectively.)

Reviewing published articles revealed that there were no proper periodic and age-specific seroepidemiological data available from Iran and most studies have reported hyperendemic patterns in their respective areas (Table 3). However, comparison between some similar older and more recent seroepidemiological studies from Mazandaran and Iran suggests a shifting pattern from high anti-HAV antibody seroprevalence to lower rates of seroprevalence (Figure 1).

**Table 1:** Demographic Characteristics and Access to Piped Drinking Water in Urban and Rural Areas of Savadkuh, Mazandaran in 2010

**Variable a**	**Urban, No. (%)**	**Rural, No. (%)**	**Total, No. (%)**	**P value**	**OR (95 % CI)**
Enrolled Subjects	506 (51.4)	478 (48.6)	984	NS	-
Male	243 (48)	232 (48.3)	475 (48.3)	NS	-
Age groups, y					
1-2.9	164	152	316 (32.1)	NS	-
3-6.9	128	126	254 (25.8)	NS	-
7-10.9	103	98	201 (20.4)	NS	-
11-17.9	59	56	115 (11.7)	NS	-
18-30	52	46	98 (9.9)	NS	-
Water supply system piped	(98)	(97)		NS	-
Educational levels b	1064	1002			
Illiterate/kindergarten	207	260	467	0.006	0.68 (0.56-0.84)
Junior school	409	512	921	< 0.0001	0.59 (0.50-0.71)
High school/diploma	371	214	585	< 0.0001	1.97 (1.64-2.40)
University	77	16	93	< 0.0001	4.8 (2.78-8.29)

^a^ All families were used enclosed defecation system in both areas there was no centralized sewage disposal system

^b^ Total numbers of 2066 is provided by 1968 parents (984 Couple) and 98 studied young adults (18 - 30 years old subjects) (984 × 2 + 98 = 2066).

**Table 2:** Anti-HAV Antibody Seroprevalence Rates by Age Groups and Residential Place in Savadkuh, Mazandaran, North of Iran in 2010

**Age Groups, y**	**Positive HAV-Antibody, No(%)**		**P value**	**OR (95% CI) a**	**Mean b**
	**Urban (n = 506)**	**Rural (n = 478)**			
1-2.9	8 (5.0)	10 (6.50)	0.51	0.46 (0.15, 1.37)	5.7
3-6.9	11 (8.6)	12 (9.50)	0.74	0.86 (0.37, 1.97)	9
7-10.9	19 (18.4)	22 (22.40)	0.48	0.80 (0.43, 1.51)	20.4
11-17.9	20 (34.0)	20 (35.7)	0.83	0.99 (0.53, 1.84)	34.8
18-30	35 (67.3)	32 (69.6)	0.81	0.93 (0.57, 1.54)	68.4
Total	93 (18.4)	96 (20.1)	0.4	0.89 (0.56, 1.23)	19.20

^a^ Seroprevalence Levels Were Increased Significantly With Age: P Value of 0.02, 0.01, 0.004, and ; Between age Group 1-2.9 Year to 18-30 Years Respectively.0.0001

^b^ Mean stands for Mean Seroprevalence Rates for Each Age Group

**Table 3:** Anti-HAV Antibody Seroprevalence Rates by the Year of Study, Age Groups, and Places (Different Provinces) of the Studies

	**Study Place**	**Year**	**Age, y**	**Prevalence, %**	**Study Subjects, No.**
**Higher Rates of Endemicity**					
Shamsizadeh A , et al. [33]	Ahwaz	2003	8-15	81.2	800
Shavaki A, et al. [36]	Azarbaijan	2006	Adults	96.5	200
Ramezani H, et al. [32]	Gazvin	2008	17-60	94.9	351
Merat S, et al. [30]	Golestan	2006	18-65	70-98.6	625
Ghadir MR, et al. [27]	Golestan	2007	25-60	70-99	697
Mohammad Alizadeh AH, et al.[31]	Hamadan	2004	10-50	85-93	171
Merat S, et al. [30]	Hormozgan	2006	18-65	70-96	453
Saffar MJ, et al. [15]	Mazandaran	1997	1-15	74.7-90.6	716
Roushan MR, et al. [23]	Mazandaran	2004	10-60	59.4-97.5	392
Alborzi P, et al. [13]	Fars	1996	2-14	33-67	-
Ehsanipour F, et al. [34]	Fars	2008	18-40	79.3-99	-
Khodaei E, et al. [14]	Tehran	1996	2-14	37-76	-
Merat S, et al. [30]	Tehran	2006	18-65	65-97	791
Ghorbani GA, et al. [28]	Tehran	2006	19-22	97.7	800
Alian S, et al.	Tehran	2006	0.6	51.7-85	1065
Ayatollahi J, et al. [26]	Yazd	2000	Adult	89.5	-
Salehi M, et al. [16]	Zabol	2000	1-15	79.6-100	229
**Lower Rates of Endemicity**					
Montazam S, et al. [21]	Azarbaijan	2005	1-15	1.2-27.2	349
Ataei B, et al. [18]	Esfahan	2005	6-65	1-28.3	816
Alian SH, et al. [17]	Mazandaran	2007	1-25	8.4-64.8	1014
Saffar MJ, et al. (this study)	Mazandaran	2010	1-30	5.7-68.4	984
Mehr AJ, et al. [20]	Tehran	2002	0.6-15	21.1-26.9	1018
Sofian M, et al. [19]	Tehran	2008	0.6-15	36.8-52.4	1056
Kazemi SA, et al. [29]	Zanjan	2005	7-10	42-45.4	300

## 5. Discussion

Current findings showed that the majority (> 65 %) of older children and adolescents remained susceptible to HAV infection. Although the education levels (as a known risk factor for HAV infection) of parents and young adults living in urban areas were significantly higher than those of rural, the seropositive rates were not statistically different between rural and urban residents (P = 0.4). These discrepancies may be explained by higher educational levels of employees working in the urban infrastructures, otherwise most other important risk factors influencing HAV transmission in a community (access to clean water, waste and sewage disposal systems, living habits and culture) were similar between residents of two studied areas.

**Figure 2:**
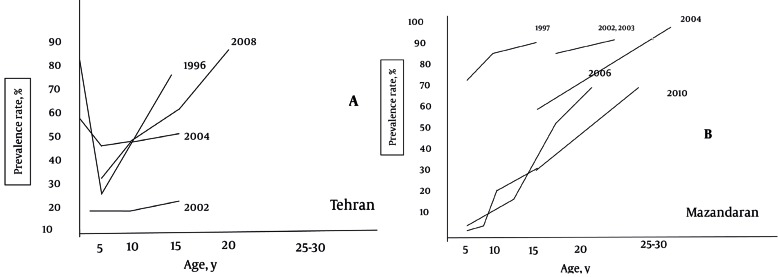
Age-Specific Seroprevalence of Hepatitis A Virus Infection in A) Tehran and B) Mazandaran From 1996 to 2010

The first published HAV epidemiological report from Mazandaran was an age-specific seroprevalence study in 1997 conducted among 1- to 15-year-old children in Sari, capital of Mazandaran province [15]. In that study high prevalence rate (87 %) of HAV infection were reported: 74.7 %, 86.7 %, and 90.6% in 1-5, 5-10, and 12-15 years old subjects, respectively. Also, hyperendemic pattern was confirmed by other studies in some special high risk groups, older children, and adults in the region [22][23][24]. However, results of a recent age-specific seroepidemiological study among 1-25-year-old population [17] demonstrated a lower infection rate especially among children, i.e. 8 %, 20 %, and 64.8 % in < 5, 5-15 and 15-25 years old individuals, respectively. When this finding was compared with data reported by earlier published studies from the region [15][22][23][24] (Figure 2A), results may suggest a declining rate of HAV infection as well as a transition to a less prevalent pattern of HAV infection. Our study findings also conforms to this concept and indicates an epidemiological shifting in Mazandaran province.

It is not easy to determine the epidemiological characteristics of the HAV infection in Iran due to lack of a nationwide database (HAV is not a reportable infection/disease except in cases of outbreaks). WHO ranked Iran among areas of high prevalence of infection with a seroprevalence of > 90 % at 10 years of age [6]. Until 1996, there was only one study published from Iran about HAV infection characteristics [25]. From 1997 to 2000, few scattered age-specific HAV seroprevalence studies were performed in some parts of the country [13][14][15][16]. Results of these studies suggested high prevalence rates of infection: 80 %, 74.7 %, 33 %, and 37 % seroimmunity levels in children < 5 years old from Zabol [16] Sari [15], Tehran [14] and Shiraz [13], respectively. These rates were increased to 100%, 90.6%, 68%, and 76% on the age of 10-15 years in those cities, respectively. Later to 2000, several seroepidemiological studies were carried out in different populations living in some parts of the country [17][18][19][20][21][22][23][24][26][27][28][29][30][31][32][33]. Results, however, were not uniform. High prevalence rates have been reported from Ahwaz [33], Hamedan [31], Tehran [14][28][34][35], Hormozgan [30], Golestan [17][27], Qazvin [32], Shiraz [13][35], Azarbaijan [36], Zanjan [29], and Yazd [26] provinces (Table 3). Other studies from Esfahan [18], Tehran, Azarbaijan [21], Mazandaran [17] demonstrated lower rates of infection especially among children (Table 3, Figure 2). These results may implicate an epidemiological transition to lower rates of infection in some parts of the country.

During past two decades, many countries in the Middle East [37][38][39][40][41] and Asia [42][43] experienced major improvement in socioeconomic status associated with urbanization, health education, access to clean drinking water, improved sanitation, and life style. Such improvements have been observed in Iran as well [44]. This pattern may reduce the rates of HAV circulation in the community especially among children, and as a result more children and adolescents remained susceptible to infection during adulthood.

Decreasing prevalence of hepatitis A infection in children in Mazandaran and some other parts of Iran, demonstrated by some recent seroprevalence studies, designates HAV as a more likely etiologic agent of acute viral hepatitis in Iran. For instance, HAV was etiologic agent of 72% and 29.6% of acute severe clinical viral hepatitis represented in two university hospitals in Zahedan [45] and Mazandaran [46] during 2003-2004 and 2002-2007, respectively.

HAV is a preventable infection by immunization. Several HAV vaccines are available that are highly effective and provide long-lasting protection above 1-2 years of age, [1][2][4][7][8][10][11]. Cost and feasibility are two major barriers of public implementation of HAV vaccination [4][7][8][47] . Recommendation on usage of HAV vaccine varies considerably among countries. Guidelines from WHO [4], CDC [7], and experts [8][10][11] on HAV vaccine emphasize on cost-benefit [47] and sustainable preventative strategies in the context of other priorities such as other diseases, and their morbidity and mortality in any given countries. Consequently, large-scale and public vaccination programs applicable in hyperendemic developing countries are not recommended. In hyperendemic countries that undergoes a lower rates of infection or intermediate endemicity, or where a large proportion of adolescents and adults remain susceptible to HAV infection and clinical hepatitis A represent a significant public health burden, large-scale childhood vaccination or targeted vaccination may be considered as a supplementary plan to health education and improved sanitation. As part of this decision process, the public health impact of HAV infection should be weighed against the impact of other vaccine-preventable infections and other preventative strategies.

In conclusion, according to the current findings and those obtained from literature review it seems that HAV epidemiology is changing in Mazandaran and Iran, and prevalence patterns are shifting to lower rates of endemicity. However, to make the most appropriate decision for pre-and post-exposure preventative strategies, periodic nationwide seroprevalence studies associated with active surveillance of clinical hepatitis A burden on public health are recommended. Until these happen, improving sanitation and health education along with targeted prophylaxis - if feasible - as a strategy for control HAV infection in Mazandaran and Iran seems to be more appropriate.
